# Pancreatic mucinous cystic neoplasm in a transgender patient

**DOI:** 10.1186/s12957-015-0620-8

**Published:** 2015-06-24

**Authors:** Deshka Foster, Mohammad F. Shaikh, Elizabeth Gleeson, Blake D. Babcock, Jianping Lin, Robert T. Ownbey, Mark E. Hysell, Daniel Ringold, Wilbur B. Bowne

**Affiliations:** Division of Surgical Oncology, Department of Surgery, Drexel University College of Medicine, 254 N. 15th St, MS 413, Philadelphia, PA 19102 USA; Department of Pathology, Drexel University College of Medicine, 254 N. 15th St, MS 435, Philadelphia, PA 19102 USA; Department of Diagnostic Radiology, Hahnemann University Hospital, 230 N. 15th St, MS 206, Philadelphia, PA 19102 USA; Department of Medicine, Drexel University College of Medicine, 254 N. 15th St, MS 427, Philadelphia, PA 19102 USA

**Keywords:** Pancreatic MCN, Transgender medicine, Hormone therapy

## Abstract

**Background:**

Cystic pancreatic lesions are increasingly more frequent detected clinical entities. Mucinous cystic neoplasm (MCN) is a hormone-related pancreatic tumor (HRTP) with a strong predominance in young and middle-aged females.

**Case presentation:**

Here, we present the case of a 31-year-old surgically transgendered female-to-male patient with a history of alcoholic pancreatitis, on chronic testosterone therapy. He was found to have a pancreatic MCN and underwent distal pancreatectomy and splenectomy.

**Conclusion:**

To our knowledge, this is the first reported case of a transgender patient with a history of hormone replacement therapy (HRT) and pancreatic MCN. We consider possible mechanisms for the pathogenesis to explain this patient’s neoplasm.

## Background

Mucinous cystic neoplasm (MCN) is a hormone-related pancreatic tumor (HRTP) with a strong predominance in young and middle-aged females [1]. In 1996, the World Health Organization and Armed Forces Institute of Pathology provided a formal distinction between intraductal papillary mucinous neoplasms (IPMN) and MCN, stressing the importance of the presence of ovarian stroma to diagnose MCN. The validity of this distinction has been confirmed in subsequent studies [2]. However, the criteria for distinguishing MCN from other mucinous tumors remain debated [2]. These neoplasms have risk for malignancy and require either close radiological follow-up or resection depending on their size and morphological characteristics [3, 4].

The exact mechanism of how sex hormones affect the development of pancreatic tumors is not entirely understood. Pancreatic tissue expresses estrogen receptors [5], and there are contributory reports of MCN development during pregnancy [6–10] and in post-menopausal women on hormone replacement therapy (HRT) [11]. There is also evidence that pancreatic tissue expresses testosterone receptors [5]. However, reports of pancreatic MCNs in males are limited and of the reports of men with MCN, most have a history of pancreatitis and/or aberrant hormone levels and sexual phenotypic characteristics [1, 12, 13]. The expression of estrogen and progesterone receptors in MCN among some male patients is perplexing [1]. There are also rare reports of pancreatic MCNs in men or post-menopausal women, which do not show the characteristic ovarian-like stroma. Some suggest that such tumors be called “indeterminate mucin-producing cystic neoplasms” so as to maintain the requirement that MCNs are defined by ovarian-like stroma [11].

Here, we present the case of a 31-year-old surgically transgendered female-to-male patient with a history of alcoholic pancreatitis, on chronic testosterone therapy. He was found to have a pancreatic MCN and underwent distal pancreatectomy and splenectomy. To our knowledge, this is the first reported case of a transgender patient on HRT with pancreatic MCN. We provide a case presentation and propose possible mechanisms, based upon our review of the literature, for the pathogenesis of this patient’s neoplasm.

## Case presentation

The patient is a 31-year-old surgically transgendered, female-to-male patient with a history of multiple prior admissions for alcoholic pancreatitis. He was on chronic, long-term testosterone therapy. He presented complaining of pain in the mid-epigastrium, which had worsened over the prior months, with occasional radiation to the back and shoulders and post-prandial worsening of pain. He denied nausea, vomiting, diarrhea, weight change, or constipation.

Additional past medical history was significant for anxiety and attention deficit hyperactivity disorder. His surgical history included laparoscopic cholecystectomy, tonsillectomy, bilateral mastectomy, total bilateral hysterectomy, and bilateral salpingo-oophorectomy. His family history included leukemia, diabetes mellitus, and cholecystitis. His medications included testosterone cypionate, bupropion, escitalopram, hyoscyamine sulfate, omeprazole, and pancrelipase. He was a former smoker with history of polysubstance abuse including chronic alcoholism.

**Fig. 1:**
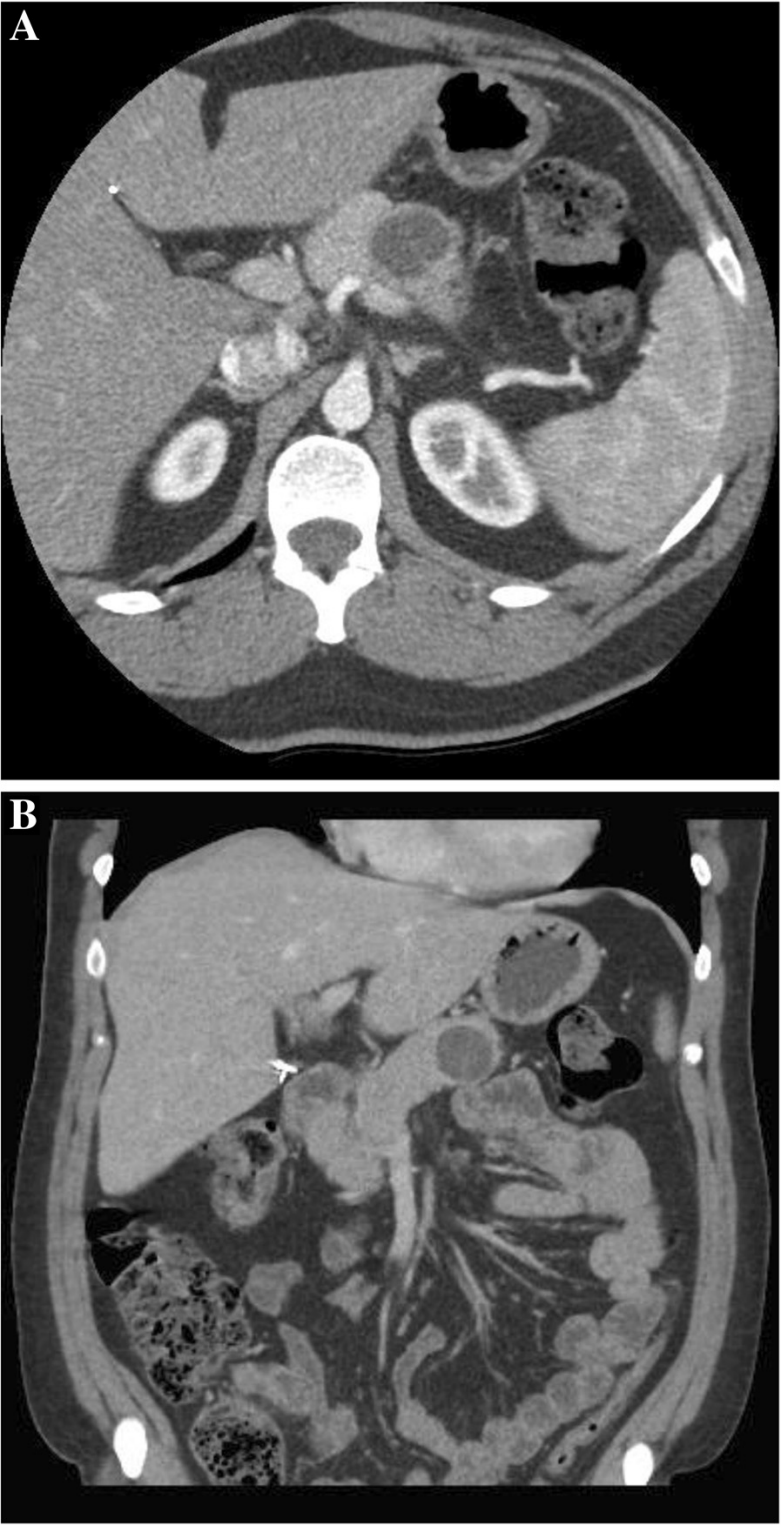
Abdominal CT with pancreatic protocol showing cystic pancreatic mass. **a** Arterial phase CT axial slice through the pancreatic body shows a cystic mass within the body region. **b** Coronal reconstruction portal venous phase shows a cystic mass confined to the pancreatic parenchyma

**Fig. 2:**
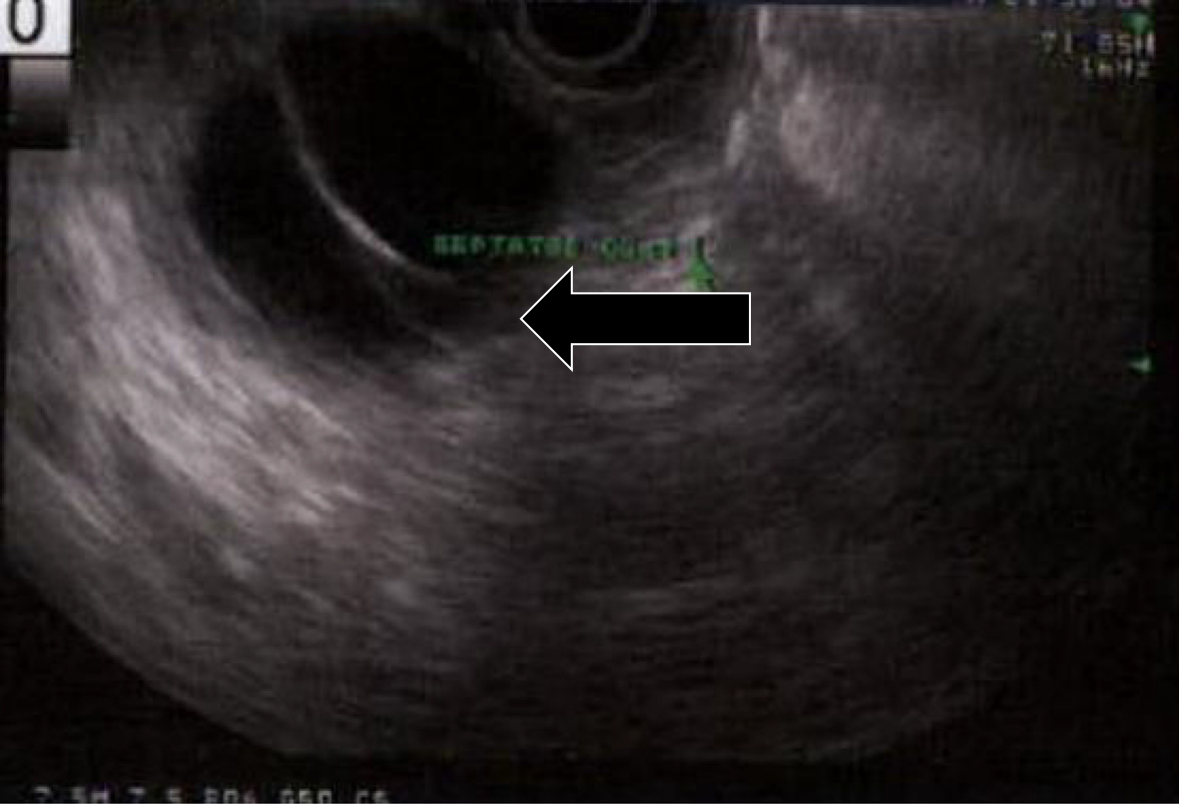
FNA-EUS showing hypo-echoic pancreatic mass, *arrow*

His physical examination was otherwise non-contributory. Serum CA19-9 level was moderately elevated at 72 u/ml [reference range <34], lipase was elevated at 128 u/l [ref 7–60], and amylase was within the normal range at 51 u/l [ref: 20–101]. Serum IgG4 was negative. Computed tomography (CT) scan of the abdomen and pelvis with pancreatic protocol demonstrated a pancreatic body cystic mass (Fig. 1a, b). Endoscopic ultrasound (EUS) confirmed a 30 × 33 mm round multi-septated mass in the pancreatic body suggestive of an MCN (Fig. 2). Aspirated cyst fluid analysis revealed an elevated CEA level of 745 ng/mL.

Of note, 6 years prior to presentation at our institution, the patient was being followed with serial cross-sectional imaging, demonstrating a pancreatic body cystic lesion. Importantly, during this interval period of time, the lesion was noted to alternately increase and decrease in size.

**Fig. 3:**
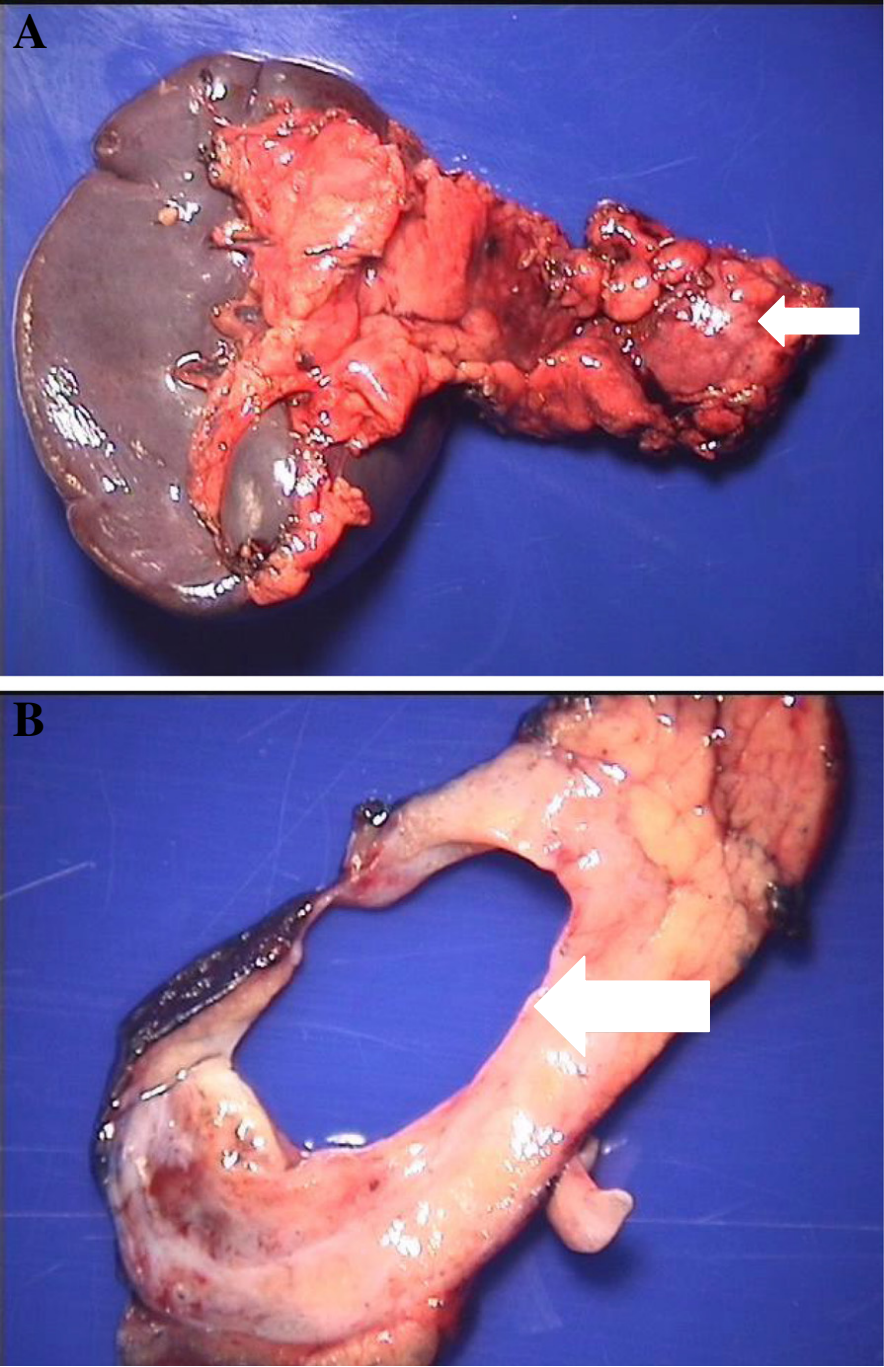
Gross pathology of the tumor. **a** A distal portion of pancreas with an attached intact spleen, *white arrow* delineates pancreatic MCN. **b** A gross section of pancreatic cyst, *white arrow* delineates pancreatic cyst wall

**Fig. 4:**
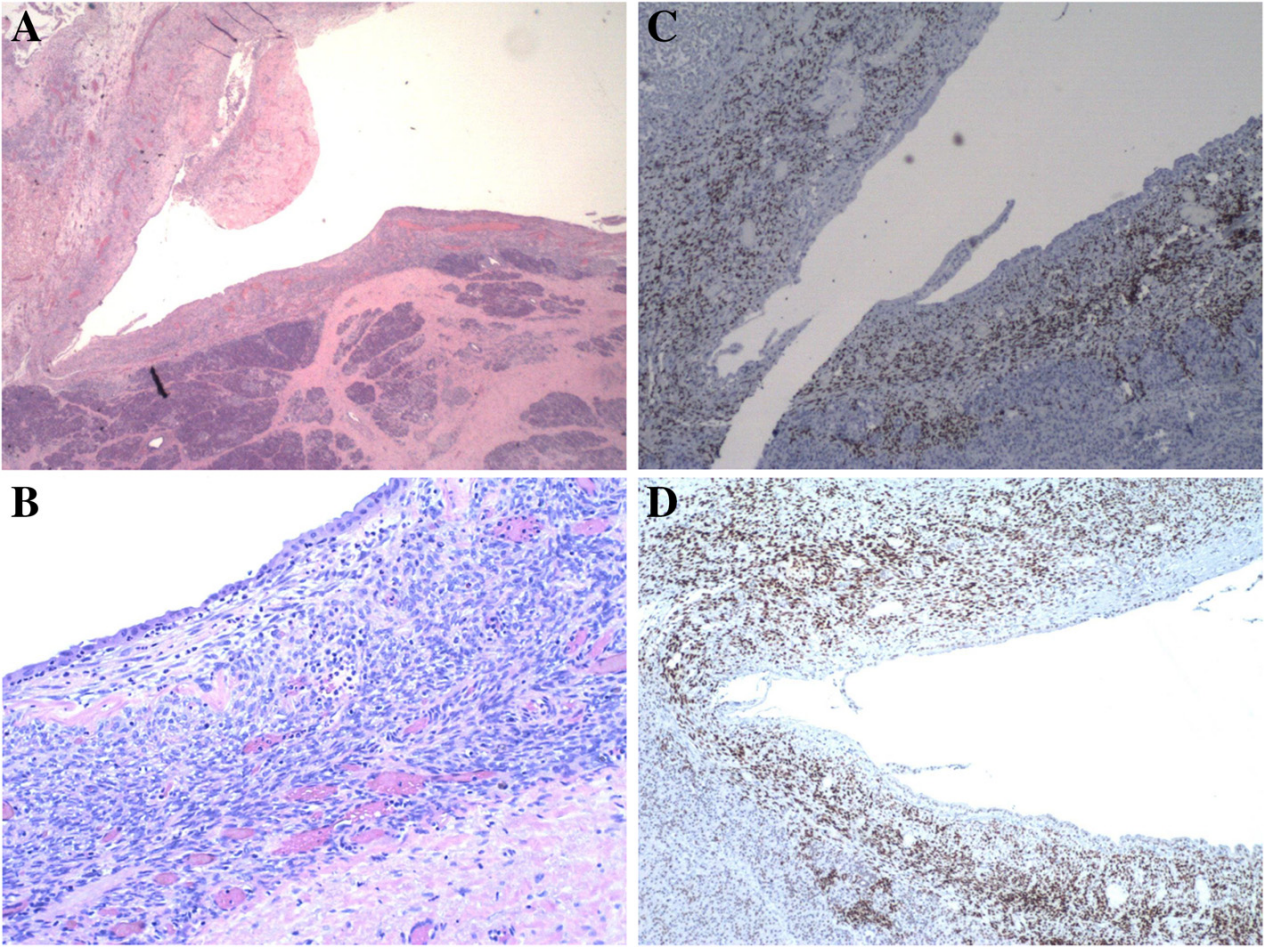
Microscopic pathology of the tumor. **a** Pancreatic MCN lined by cuboidal epithelium surrounded by the ovarian-like stroma (H&E stain; **c** × 1.25). **b** High-power of pancreatic MCN lined by cuboidal epithelium surrounded by the ovarian-like stroma (H&E stain; **d** × 10). **c** Immunohistochemical staining for estrogen receptor within the MCN stroma (4×). **d** Immunohistochemical staining for progesterone receptor within the MCN stroma (4×)

Surgical management included distal pancreatectomy and splenectomy. Specimens were sent for pathologic examination. Serial sectioning of the formalin-fixed specimen revealed a 2 × 1.8 × 1.2 cm septated cyst with a tan-pink, smooth, and glistening inner lining, filled with clear, thin fluid. The cyst was located 0.9 cm from the proximal pancreatic resection margin, 6.7 cm from the splenic hilum, and 0.1 cm from the nearest posterior aspect. It abutted but did not involve the main pancreatic duct (Fig. 3a, b). Microscopic examination showed that the cyst was lined by cuboidal or mucin-producing epithelium and surrounded by the ovarian-like stroma, which was immunohistochemically positive for estrogen and progesterone receptors (Fig. 4a–d). These characteristics are consistent with the diagnosis of MCN. The patient’s post-operative course was complicated by a peri-pancreatic abscess and fistula, which resolved following endoscopic placement of a pancreatic ductal stent. On yearly follow-up, the patient had no evidence of recurrent disease.

## Discussion

**Fig. 5:**
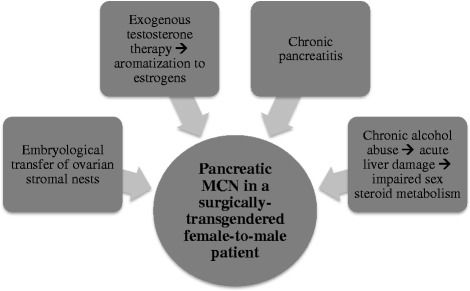
Proposed multifactorial mechanisms for pathogenesis of pancreatic MCN in a transgender male

Pancreatic MCN is a HRTP with predominance in females [1], characterized by ovarian-like stroma [4]. Figure 5 outlines the possible mechanisms for the pathogenesis of pancreatic MCN in our patient, which is likely multifactorial. Investigators have speculated about the interaction between HRT and the development of hormone-related cancers for many years. Among transgendered females, there are several case reports describing the occurrence of prostate cancer after many decades of estrogen therapy [14, 15]. The exact role that estrogen plays in the development of pancreatic MCN is not entirely understood [16]. However, the pancreas is known to express estrogen receptors and binding proteins [5]. Normal pancreatic tissue can convert estrone and estrone sulfate into active 17-beta-estradiol, and estrogen is believed to be necessary for pancreatic enzyme synthesis [5]. There are contributory reports of MCN development during pregnancy [6–10] and in post-menopausal women on HRT [11].

One explanation for the pathogenesis of pancreatic MCN expressing ovarian-like stroma is that genital gland and dorsal pancreatic buds are adjacent during the course of development such that primary yolk cells may be implanted in the pancreas [16]. This mechanism is plausible in the patient presented here. Another explanation is that immature pancreatic stroma, which expresses sex hormone receptors, responds to female hormones resulting in cystic change and progression [16]. In either case, estrogen is required to stimulate tissue proliferation. The patient discussed here is female-to-male transgendered on chronic testosterone therapy. It is plausible that exogenous testosterone could have been aromatized to estradiol. This would be particularly true if the testosterone dosing was relatively high. Aromatase and 5-alpha-reductase have both been identified in human pancreatic cancer tissue [17]. The former enzyme converts testosterone to 5-dihydrotestosterone, which is a strong androgen, and the latter enzyme converts testosterone to estradiol as well as delta-4-androstenedione to estrone. Importantly, notable variations in size of our patient’s pancreatic MCN may reflect variation in the hormonal environment from estrogen to testosterone, which lends credence to the hypothesis of hormonal influences on MCN development and natural history.

The aforementioned embryological mechanism, however, does not fully account for the albeit rare occurrence of pancreatic MCN in men [1, 12]. The literature concerning pancreatic MCN in males is limited, and it is possible that the development of MCN in men may not necessarily follow the same pathogenesis as occurs in women [12]. However, of the reports of men with MCN, most have a history of pancreatitis and/or aberrant hormone levels and sexual phenotype characteristics [1, 12, 13]. Moreover, in a recent study that included five cases of pancreatic MCN along with four cases of pancreatic solid pseudopapillary neoplasm (SPN) in men, hormonal and/or sexual dysfunction was identified in four of the nine patients [13]. In one case report of a young Japanese man who was found to have a pancreatic MCN with ovarian-type stroma and antiestrogen and antiprogesterone staining positivity, the authors question whether or not the presence of female hormone receptors is adequate to term the tissue of origin to be ovarian. Pancreatic tissue is also known to express testosterone receptors as well as estrogen receptors [5]. It has been established that there are androgen receptors in pancreatic cancer, and testosterone has been experimentally shown to promote growth of pancreatic adenocarcinoma [5].

Chronic pancreatitis is another proposed risk factor for the development of pancreatic MCN. Wouters et al. published a case report discussing a 43-year-old man who had a 10-year history of moderate to high alcohol intake and chronic pancreatitis that developed a pancreatic MCN [1]. Accordingly, a possible mechanism for the development of pancreatic MCN in chronic alcohol abusers, male or female, is chronic excess alcohol consumption that results in increased estrogen levels either via increased peripheral fat conversion of androgen precursors to estrogen and/or via transient or permanent liver damage resulting in hormonal imbalance secondary to disrupted sex steroid metabolic pathways with elevated estrogen levels [1]. The patient presented in this case had a long history of alcohol abuse.

In terms of management, surgical resection remains the cornerstone for the treatment of high risk pancreatic cystic lesions. Clinicopathologic factors identified as higher risk for malignancy among pancreatic cystic neoplasms includes size greater than 3 cm, presence of septations, a solid component, and related symptomatology [3, 4].

## Conclusion

Here, we present the first reported case of a pancreatic MCN in a surgically transgendered female-to-male patient. This patient had a history of alcoholic pancreatitis and was on chronic, long-term testosterone therapy. We provide a case presentation and propose possible mechanisms, based upon our review of the literature that could explain the pathogenesis of his neoplasm. Transgendered patients generally tolerate HRT well. However, this case suggests that pancreatic neoplasms, in part, may be susceptible to HRT. This case also illustrates the importance of being aware of special medical and surgical issues in the transgender population.

## Consent

Informed consent was obtained from the patient for publication of this case report and any accompanying images.

## Abbreviations

CT: computed tomography
EUS: endoscopic ultrasound
HRT: hormone replacement therapy
HRTP: hormone-related pancreatic tumor
IPMN: intraductal papillary mucinous neoplasms
MCN: mucinous cystic neoplasm
SPN: solid pseudopapillary neoplasm
